# MR diagnosis of SCC arising within ovarian cystic teratomas: analysis of mural nodule characteristics

**DOI:** 10.1007/s00261-024-04186-y

**Published:** 2024-02-23

**Authors:** Takuya Fukuzawa, Ayumi Ohya, Mika Tanaka, Marika Shimizu, Kentaro Kobayashi, Tomohito Matsushita, Tomofumi Watanabe, Hisanori Kobara, Yasunari Fujinaga

**Affiliations:** 1 Department of Radiology, Shinshu University School of Medicine, 3-1-1 Asahi, Matsumoto, 390-8621 Japan; 2 Department of Radiology, Fujimi Kogen Hospital, 11100 Ochiai, Fujimi-Cho, Suwa-Gun, 399-0214 Japan; 3 Department of Radiology, Iida Municipal Hospital, 438 Yawatamachi, Iida, 395-8502 Japan; 4 Department of Obstetrics and Gynecology, Shinshu University School of Medicine, 3-1-1 Asahi, Matsumoto, 390-8621 Japan

**Keywords:** Ovarian teratoma, Squamous cell carcinoma, Cysts, Retrospective studies, Magnetic resonance imaging

## Abstract

**Purpose:**

This study aims to evaluate and identify magnetic resonance (MR) findings of mural nodules to detect squamous cell carcinoma arising from ovarian mature cystic teratoma (SCC-MCT).

**Methods:**

This retrospective study examined 135 patients (SCC-MCTs, *n* = 12; and benign MCTs, *n* = 123) with confirmed diagnoses across five different institutions between January 2010 and June 2022. Preoperative MR images for each patient were independently assessed by two experienced radiologists and analyzed following previously reported findings (PRFs): age, tumor size, presence of mural nodules, size of mural nodule, and the angle between mural nodule and cyst wall (acute or obtuse). Furthermore, this study evaluated four mural nodule features—diffusion restriction, fat intensity, Palm tree appearance, and calcification—and the presence of transmural extension.

**Results:**

There were significant differences between the SCC-MCT and benign MCT groups in terms of all PRFs and all mural nodule findings (*p* < 0.01). Among the PRFs, “tumor size” demonstrated the highest diagnostic performance, with a sensitivity of 83.3% and a specificity of 88.6%. A combination of the aforementioned four mural nodule findings showed a sensitivity and specificity of 83.3% and 97.6%, respectively, for the diagnosis of SCC-MCT. Regarding diagnosis based on a combination of four mural nodule findings, the specificity was significantly higher than the diagnosis based on tumor size (*p* = 0.021). Based on these mural nodule findings, three SCC-MCT patients without transmural invasion could be diagnosed.

**Conclusion:**

Mural nodule MR findings had a higher diagnostic performance than PRFs for SCC-MCT and can potentially allow early detection of SCC-MCTs.

**Graphical abstract:**

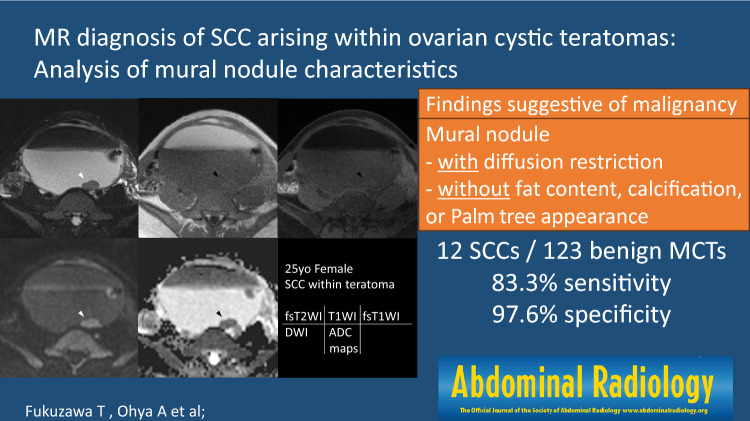

## Introduction

Mature cystic teratoma (MCT) is a common, benign ovarian neoplasm, accounting for 20% of all ovarian neoplasms and 60% of all benign ovarian tumors [1, 2]. Malignant transformation occurs in approximately 2% of MCTs (range, 0.8%–5.5%) [2], with squamous cell carcinoma (SCC) being the most common type, accounting for 80% of the reported cases [1]. SCC arising in MCT (SCC-MCT) has a significantly worse outcome compared to common epithelial ovarian cancers, with a 5-year overall survival rate ranging from 28% to 66.7% [3, 4]. Tumor stage and debulking status are the strongest prognostic factors of SCC-MCT [1, 3, 5]. Reportedly, patients with SCC-MCTs tend to be older and have larger tumors compared to those with benign MCTs [1, 5]. Moreover, SCC-MCTs often present with transmural extension with an obtuse angle between the mural nodules and the cyst wall on imaging [3, 6, 7]. However, early detection of this malignancy is challenging, and the diagnostic accuracy of ultrasound, magnetic resonance (MR) imaging, and computed tomography (CT) continue to remain a topic of debate [2, 4, 7, 8]. Moreover, a diagnosis of early-stage SCC-MCT is often accidentally made during an operation or on final histological examination [4].

MCT is composed of mature tissue that originates from two or three germ layers—ectoderm, mesoderm, and endoderm [1, 9, 10]. The tumor wall predominantly consists of tissues derived from the ectoderm and mesoderm—such as skin and hair (ectoderm-derived tissues); fat and muscle (mesoderm-derived tissues); and other components, including glial tissue, thyroid tissue, fibrous stroma, or vessels [8, 10]. On MR imaging, MCT typically presents as a cystic lesion with fat content, often accompanied with benign mural nodules, known as Rokitansky nodules or dermoid plugs. These nodules contain fat, exhibit a Palm tree appearance, and demonstrate calcification, reflecting their diverse tissue composition and various signal intensities [2, 7, 8, 10–12]. Moreover, SCC-MCT pathologically presents as nodular components inside the cystic tumor or as plaques on the cyst wall [2, 7] and can be distinguished pathologically from the mural nodules observed in benign MCTs. Therefore, we speculate that MR findings differ between benign MCTs and SCC-MCTs, owing to differences in pathological characteristics. Furthermore, since SCC is typically associated with diffusion restriction on MR imaging, we hypothesized that the findings suggestive of malignancy in the teratoma would be as follows: the mural nodule would exhibit diffusion restriction but lack fat, calcification, or Palm tree appearance, all of which are characteristic of Rokitansky nodules. No study to date has assessed detailed MR features of mural nodules in SCC-MCTs. Consequently, in this study, we aim to elucidate characteristic MR findings of mural nodules in SCC-MCTs. We also aim to evaluate the diagnostic performance of the characteristic MR findings of mural nodules compared to previously reported tumor and mural nodule findings.

## Methods

### Patient population

This study was collectively reviewed and approved by our institutional ethical committee (no. 5672). The need for informed consent was waived owing to the retrospective nature of the study. Between January 2010 and June 2022, patients diagnosed with SCC-MCT were identified via electronic medical records at our institution and following four other institutions: Nagano Red Cross Hospital, Shinonoi General Hospital, Suwa Red Cross Hospital, and Iida Municipal Hospital. We reviewed the pathology database of our hospital and selected patients using the keywords “ovary” and “mature teratoma” and “squamous cell carcinoma.” Patients were selected in a similar manner at other institutions. The inclusion criteria were as follows: patients with SCC-MCT treated surgically; those that underwent preoperative MR imaging including diffusion-weighted imaging (DWI) with apparent diffusion coefficient (ADC) maps; and those that had been provided a final pathological diagnosis. Consequently, 12 patients (median age, 51 years; range, 25–69 years) were classified into the SCC-MCT group. We re-searched the pathology reports in our institution during the same investigation period using keywords “ovary” and “mature teratoma” and excluded cases with malignant transformation manually. Through this process, 123 patients were identified who were provided a pathological diagnosis of benign MCTs and underwent preoperative MR imaging and surgical resections. These patients were classified into the benign MCT (b-MCT) group.

### MR image condition and analysis

**Fig. 1:**
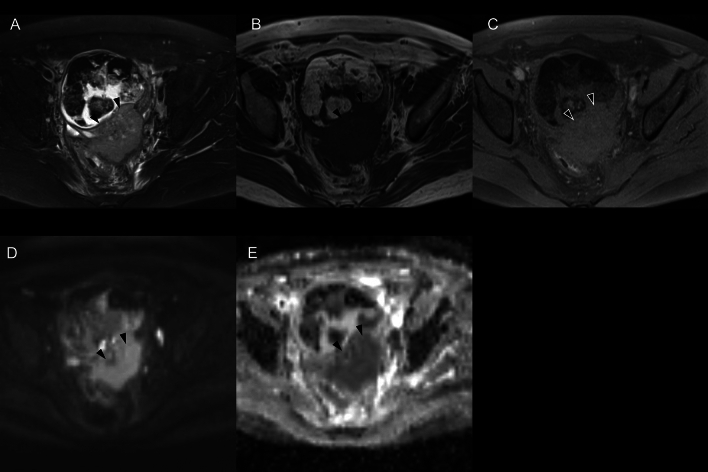
Squamous cell carcinoma arising in ovarian mature cystic teratoma with transmural extension in a 53-year-old woman. **a** Axial fat-suppressed T2-weighted image (T2WI) shows a cystic lesion in the pelvis with a solid component (arrowhead). The solid component protrudes from the wall of the cyst toward the dorsal side, suggesting transmural extension and does not have any fat signal or calcification. **b**, **c** Axial T1-weighted image (T1WI) and fat-suppressed T1WI reveal hyperintensity in the cystic component on T1WI, which changes to hypointensity on fat-suppressed T1WI, confirming the presence of fat. **d**, **e** The solid component shows hyperintensity on diffusion-weighted images and is hypointense on apparent diffusion coefficient maps

**Fig. 2:**
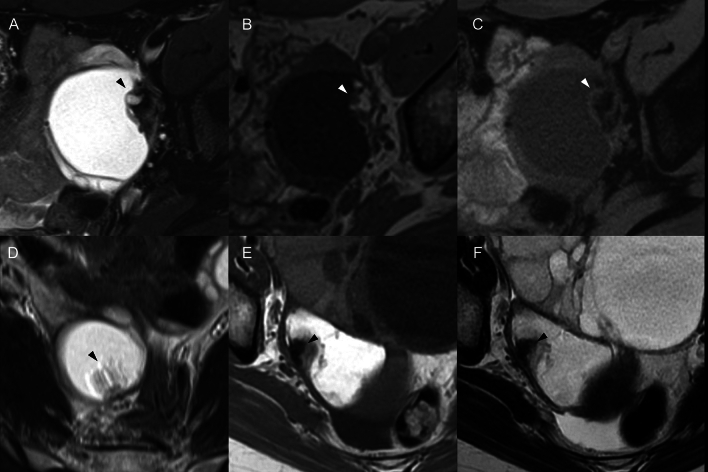
A mural nodule in benign mature cystic teratoma. **a**–**c** A 27-year-old woman. Axial fat-suppressed T2-weighted image (T2WI) **a** shows a hyperintense mass with a heterogeneous mural nodule along the left side wall of the mass (arrowhead), axial T1-weighted image (T1WI) **b** exhibits the mural nodule with internal hyperintensity, and on axial fat-suppressed T1WI **c**, the hyperintensity within the mural nodule on T1WI changes to a hypointensity, indicating a fat component. **d** A 28-year-old woman. An axial T2WI displays a palm tree-like structure projecting on the hyperintense mass (arrowhead). **e**, **f** A 36-year-old woman. Axial T1WI **e** and T2WI **f** reveal a signal void indicative of a tooth-like structure

All MR images were obtained prior to surgery. The median days between preoperative MR imaging and surgical resection were 48 days (range, 7–127 days) in the SCC-MCT group. In the benign MCT (b-MCT) group, the median duration was 98 days (range, 0–485 days). The scan parameters of MR images varied because this was a retrospective, long-term, and multicenter study. All images were assessed by two radiologists with 7 and 20 years of experience. The radiologists evaluated T1- and T2-weighted images, corresponding fat-suppressed images, and DWIs with ADC maps. Without referring to the patient, clinical, and pathological information, the two radiologists independently assessed the MR images of the lesions, with a focus on the following MR findings: maximum tumor size, presence of mural nodules, and presence of transmural extension. The maximum diameter on a MR image was measured using the axial T2-weighted images. Nodules of ≥ 3 mm in size or solid components in the cyst were defined as mural nodules (Figs. 1 and 2). If mural nodules were present, the radiologists investigated the characteristics of all mural nodules in the tumor, focusing on the following MR findings: size, angle formed with the cyst wall (acute or obtuse), and presence/absence of diffusion restriction, fat content, Palm tree appearance, and calcification. The angle with the cyst wall was measured using an axial T2-weighted image. Two angles formed by the cyst wall and the mural nodule were measured and were defined as “obtuse” if both angles were > 90 degrees. The intensity of each nodule on DWI and ADC maps were compared to the gluteus maximus muscle and were graded as hyperintensity, isointensity, or hypointensity. The lesion was assumed to have a diffusion restriction when it was hyperintense on DWI and hypointense on the corresponding ADC maps (Fig. 1d, e). The fat content within mural nodule were evaluated on fat- and non-fat-suppressed images (Fig. 2b, c). The Palm tree appearance was classified as positive in cases with palm tree-like masses projecting into the cyst cavity on T2-weighted images (Fig. 2d) [13]. A marked hypointense area or signal void on both T1- and T2-weighted images within the mural nodule was considered a calcification (Fig. 2e, f). Transmural invasion was defined as solid tissue spreading beyond the cyst wall into the surrounding tissue (Fig. 1). When multiple teratomas were present in one patient, the largest lesion was selected for analysis. When multiple mural nodules were observed within the tumor, the mural nodule with the greatest number of findings suggestive of malignancy—i.e., exhibiting diffusion restriction and lacking fat content, Palm tree appearance, and calcification—was selected and defined as the “most worrisome mural nodule.” In cases where multiple nodules presented with an equal number of these features, the largest nodule was chosen for evaluation. Any disagreements between the two radiologists were resolved by discussion until a consensus was reached.

### Statistical analysis

**Fig. 3:**
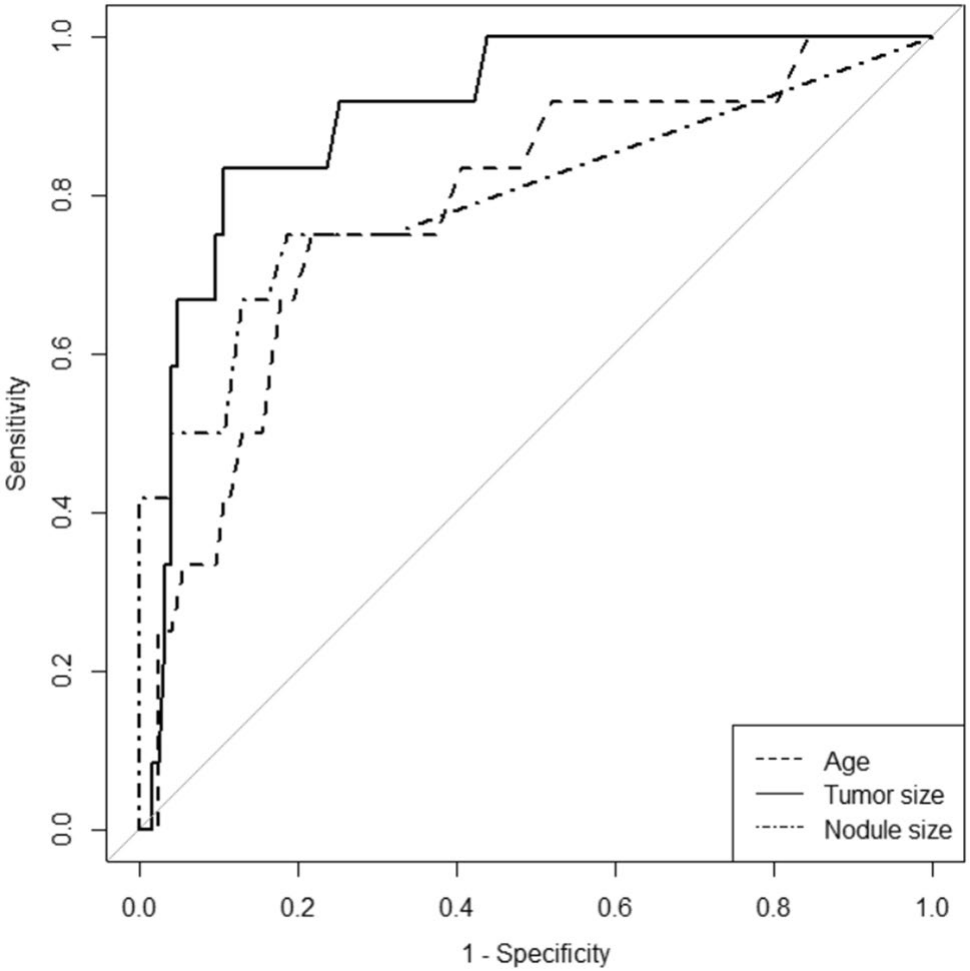
Receiver operating characteristic curve of the previously reported findings. The area under the curve values of the parameters of age, tumor size, and nodule size were 0.78 (95% CI 0.64–0.93), 0.90 (0.83– 0.98), and 0.85 (0.71–0.99), respectively

We constructed receiver operating characteristic (ROC) curves for the diagnosis of SCC-MCT based on age, tumor size, and mural nodule size. In cases without mural nodules, the diameter of the mural nodules was calculated as 0 mm. We calculated the area under the curve (AUC) and the cut-off values at which the Youden index reached its maximum (Fig. 3). Furthermore, we calculated the sensitivity and specificity of each MR finding to differentiate between SCC-MCT and b-MCT. The Wilcoxon rank sum test was used to compare continuous variables, such as patient age and maximum lesion diameter, between the SCC-MCT and b-MCT groups. In the evaluation of mural nodules, specific criteria were employed for interpretation. For example, when determining the presence of diffusion restriction in mural nodules, the following classification was adopted: Cases without mural nodules, as well as those with mural nodules but not exhibiting diffusion restriction, were categorized as “Negative.” On the other hand, cases were considered “Positive” only if the mural nodules demonstrated diffusion restriction. We used Fisher’s exact test to compare binary variables, such as the presence or absence of mural nodule, diffusion restriction, fat intensity, Palm tree appearance, and calcification within mural nodules between the two groups. Finally, we used the McNemar mid-*p* test to compare sensitivity and specificity. *p* < 0.05 was considered statistically significant. To evaluate the inter-reader agreement of mural nodule findings between the two radiologists, Cohen’s kappa coefficient was calculated. A kappa value of ≤ 0.20 indicated poor agreement; 0.21–0.40, fair agreement; 0.41–0.60, moderate agreement; 0.61–0.80, good agreement; and 0.81–1.00, excellent agreement. All analyses were performed using R software, version 3.6.2 [14].

## Results

Characteristics of the SCC-MCT group are presented in Table 1. Mural nodules were observed in 10 of 12 patients. All of the “most concerning or worrisome mural nodules” in SCC-MCTs were those that met the criteria for findings suggestive of malignancy, i.e., each nodule exhibited diffusion restriction and lacked fat content, calcification, and Palm tree appearance characteristic of Rokitansky nodules.

**Table 1:** MR findings of SCC-MCT group

No	Age (years)	MR findings									Transmural invasion
		Size (mm)	Nodule findings								
			No. of nodule	Findings of most worrisome nodule							
				Angle	Size (mm)	DR	FC	PA	Cal	FSM	
1	39	143	1	Obtuse	76	P	N	N	N	P	P
2	69	138	1	Obtuse	68	P	N	N	N	P	P
3	49	151	2	Obtuse	63	P	N	N	N	P	P
4	53	106	1	Obtuse	61	P	N	N	N	P	P
5	64	139	2	Obtuse	60	P	N	N	N	P	P
6	69	59	1	Obtuse	37	P	N	N	N	P	P
7	49	104	1	Obtuse	28	P	N	N	N	P	P
8	55	79	1	Obtuse	23	P	N	N	N	P	N
9	25	155	2	Acute	23	P	N	N	N	P	N
10	47	155	2	Acute	18	P	N	N	N	P	N
11	35	122	0	–	–	–	–	–	–	–	N
12	69	174	0	–	–	–	–	–	–	–	N

When multiple mural nodules were observed within the tumor, the most worrisome mural nodule was defined as the one with the greatest number of characteristics indicative of FSM

*MR* magnetic resonance, *SCC-MCT* squamous cell carcinoma arising in mature cystic teratoma, *Angle* between nodule and wall of the cyst, *DR* diffusion restriction, *FC* fat content, *PA* Palm tree appearance, *Cal* calcification, *FSM* findings suggestive of malignancy (mural nodule with diffusion restriction and without fat content, calcification, or Palm tree appearance, *P* positive, *N* negative, – not available

**Fig. 4:**
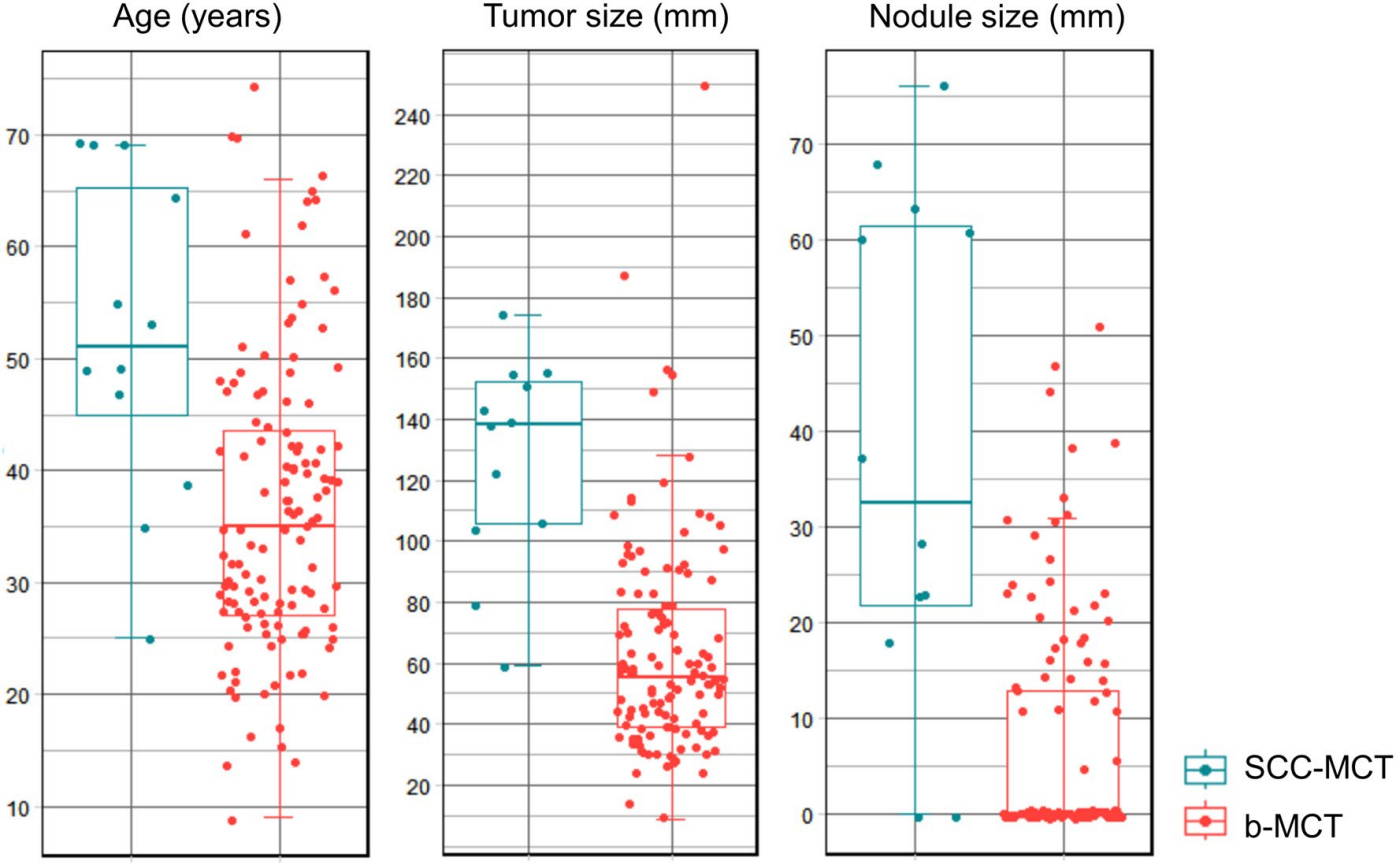
Boxplots of age, tumor size, and nodule size of patients with SCC-MCT and benign MCT. In all findings, overlap of values is seen in the two groups. *MCT* mature cystic teratoma, *SCC* squamous cell carcinoma

We also confirmed the diagnostic ability of previously identified previously reported findings (PRFs) as follows. The patients in the SCC-MCT group were significantly older than those in the b-MCT group (*p* < 0.01) (Table 2). The tumor and mural nodule size in the SCC-MCT group was significantly larger than that in the b-MCT group (*p* < 0.001) (Table 2). The frequency of mural nodules and number of nodules forming obtuse angles with the wall was significantly higher in the SCC-MCT group (*p* < 0.001) (Table 2). Transmural extension was observed in seven of 12 patients in the SCC-MCT group, but was not observed in the b-MCT group. The AUC values of age, tumor size, and nodule size were 0.78 (95% confidence interval [CI] 0.64–0.93), 0.90 (0.83–0.98), and 0.85 (0.71–0.99), respectively (Fig. 3). The cut-off values for age, tumor size, and nodule size that maximized the Youden index were 46 years, 103 mm, and 17 mm, respectively. Even at these cut-off values of PRFs, many cases in the benign MCT group overlapped with those of the SCC-MCT group (Fig. 4).

**Table 2:** Characteristics of the two groups

	SCC-MCT (*n* = 12)	b-MCT (*n* = 123)	*p* value
Age (years)	51 [25–69]*	35 [9–74]*	0.0012^a^
Cyst size (mm)	139 [59–174]*	55 [9–249]*	< 0.001^a^
Mural nodule present	83.3% (10/12)	31.7% (39/123)	< 0.001^b^
Nodule size (mm)	30 [0–76]*	0 [0–51]*	< 0.001^a^
Obtuse angle between cyst wall and nodule	66.7% (8/12)	4.1% (5/123)	< 0.001^b^
Transmural invasion	58.3% (7/12)	0% (0/123)	< 0.001^b^

*SCC-MCT* squamous cell carcinoma arising in mature cystic teratoma, *b-MCT* benign mature cystic teratoma

*median (range)

^a^Wilcoxon rank sum test was performed

^b^Fisher’s exact test was performed

Diagnostic performance of each MR finding for nodules is presented in Table 3. The sensitivity and specificity of SCC-MCT diagnosis using the aforementioned cut-off values were as follows: age, 75.0% (95% CI 42.8–94.5%) and 76.4% (67.9–83.6%), respectively; tumor size, 83.3% (51.6–97.9%) and 88.6% (81.6–93.6%), respectively; and nodule size, 83.3% (51.6–97.9%) and 81.3% (73.3–87.8%), respectively. Tumor size had the highest diagnostic performance among conventional MR findings. In contrast, the sensitivity and specificity using the findings suggestive of malignancy were 83.3% (95% CI 51.6–97.9%) and 97.6% (93.0–99.5%), respectively. The inter-reader agreement between the two radiologists for findings suggestive of malignancy was excellent, with Cohen’s kappa coefficients being 0.86. This specificity was significantly higher than that using the previously mentioned cut-off values for age, tumor size, and nodule size (*p* < 0.01).

**Table 3:** Diagnostic performance of each MR finding

	Cut-off value	Sensitivity (95% CI)	Specificity (95% CI)
Previously reported findings			
Age	46 years	75.0% (42.8–94.5%)	76.4%^b^ (67.9–83.6%)
Tumor size	103 mm	83.3% (51.6–97.9%)	88.6%^a^ (81.6–93.6%)
Mural nodule size	17 mm	83.3% (51.6–97.9%)	81.3%^b^ (73.3–87.8%)
Obtuse angle between cyst wall and mural Nodule		66.7% (34.9–90.1%)	95.9% (90.8–98.7%)
Transmural invasion		58.3% (27.7–84.8%)	100.0% (95.6–100.0%)
Nodule findings			
Nodule with diffusion restriction		83.3% (51.6–97.9%)	80.5% (72.4–87.1%)
Nodule without fat content		83.3% (51.6–97.9%)	90.2% (83.6–94.9%)
Nodule without calcification		83.3% (51.6–97.9%)	73.2% (64.4–80.8%)
Nodule without Palm tree appearance		83.3% (51.6–97.9%)	81.3% (73.3–87.8%)
FSM		83.3% (51.6–97.9%)	97.6% (93.0–99.5%)

*FSM*, findings suggestive of malignancy (mural nodule with diffusion restriction and without fat content, calcification, or Palm tree appearance)

^a^The specificity of FSM was significantly higher than that of the previously reported findings, such as tumor size ≥ 103 mm (*p* < 0.01^a^), age over 46 years (*p* < 0.001^b^), and nodule size ≥ 17 mm (*p* < 0.001^b^)

There was no significant difference in sensitivity between “FSM” and any previously reported finding

**Fig. 5:**
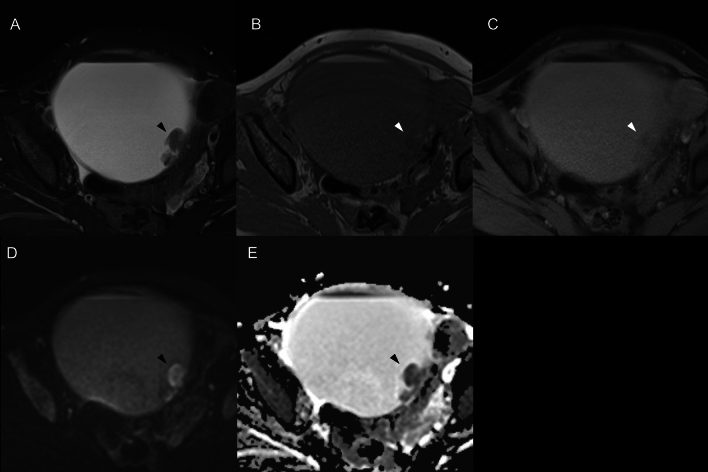
A 47-year-old woman with squamous cell carcinoma arising in an ovarian cystic teratoma without transmural extension. MR images reveal mural nodule consistent with “findings suggestive of malignancy” (mural nodule with diffusion restriction and without fat content, calcification, and Palm tree appearance). **a** Axial fat-suppressed T2-weighted image shows a 155 mm-sized large cystic lesion in the pelvis with typical fat-fluid level. The mural nodule (arrowhead) is located on the left side of cystic wall, forming an acute angle with the cystic wall and does not exhibit the Palm tree appearance. **b** Axial T1-weighted in-phase image could not demonstrate the mural nodule clearly. **c** Axial T1-weighted fat-suppressed images do not show the signal drop of the nodule. The solid nodule does not exhibit any strong hypointense areas suggestive of calcification. **d**, **e** The mural nodule shows hyperintensity on diffusion-weighted images and hypointensity on apparent diffusion coefficient maps, suggesting restricted diffusion

**Fig. 6:**
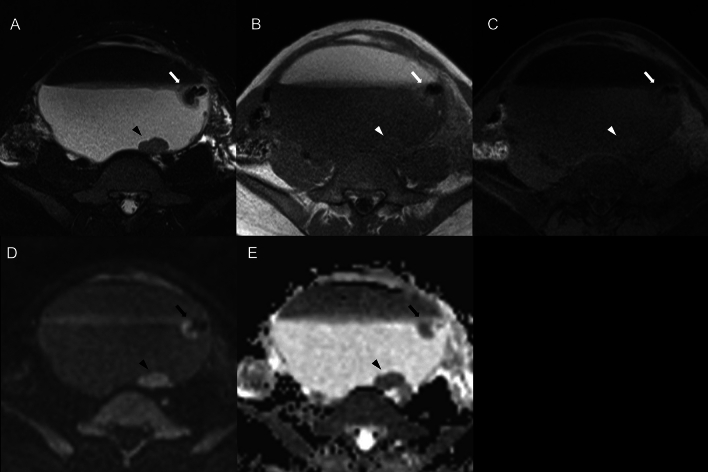
A 25-year-old woman with squamous cell carcinoma arising in an ovarian cystic teratoma without transmural extension. MR images show two distinct mural nodules: one consistent with “findings suggestive of malignancy” (mural nodule with diffusion restriction and without fat content, calcification, or Palm tree appearance) (arrowhead) and another exhibiting calcification and fat content (arrow). **a** Axial fat-suppressed T2-weighted image reveals a 155 mm-sized large cystic lesion in the pelvis. The nodule located dorsally in the cyst (arrowhead) shows isointensity, forming an acute angle with the cystic wall, while the nodule on the left side wall of the cyst (arrow) has strong hypointense areas suggestive of calcification. Neither nodule shows a Palm tree appearance. **b**, **c** In axial T1-weighted in-phase and fat-suppressed images, the nodule located dorsally in the cyst (arrowhead) is not clearly delineated. Conversely, the nodule on the left side wall of the cyst (arrow) presents areas of signal drop in the fat-suppressed images, indicating the presence of fat. **d**, **e** Axial diffusion-weighted images and ADC maps show that the nodule located dorsally in the cyst (arrowhead) has diffusion restriction. In contrast, the nodule on the left side wall of the cyst (arrow) has areas believed to be fat, with no other signs of diffusion restriction

**Fig. 7:**
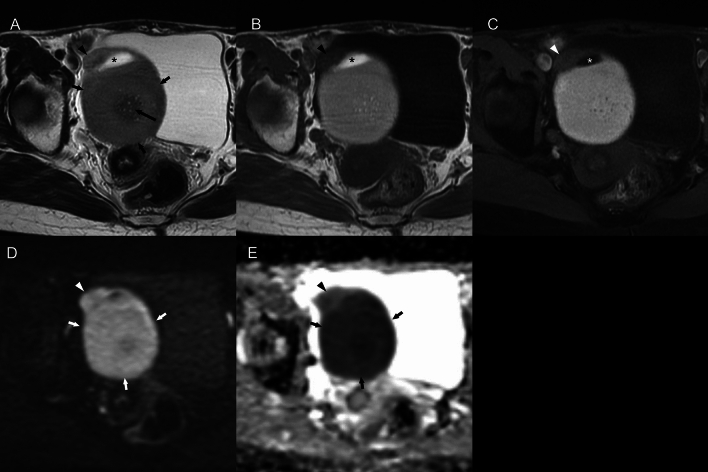
A 55-year-old woman with squamous cell carcinoma arising in an ovarian cystic teratoma without transmural extension, shows a mural nodule on MR images consistent with “findings suggestive of malignancy” (mural nodule with diffusion restriction and without fat content, calcification, or Palm tree appearance). **a** Axial fat-suppressed T2-weighted image reveals a 79 mm-sized cystic lesion in the pelvis. The mural nodule (arrowhead) protruded from of the ventral cyst wall does not exhibit a Palm tree appearance and forms an obtuse angle with the cystic wall. While the nodule also protrudes outward from the wall, its margins are smooth, and there are no findings suggestive of transmural invasion. There is a hyperintensity area adjacent to the mural nodule suggesting fat component (*). The dorsal cystic portion was shown as a slightly hyperintense area (short arrows). A hypointense area in the dorsal cystic portion suggests hair ball (long arrow). **b**, **c** Axial T1-weighted and fat-suppressed images do not show any distinct fatty signals in the nodule (arrowhead) though there is a fat component (*) adjacent to the mural nodule. The dorsal cystic portion was shown as a hyperintense area on T1-weighted images with/without fat-suppression (short arrows). **d**, **e** An axial diffusion-weighted images and an apparent diffusion coefficient (ADC) map indicate restricted diffusion within the nodule (arrowhead). Most of the part of the dorsal cystic portion is also shown as diffusion restriction, suggesting it contains the mixture of keratin and sebum (short arrows)

Notably, the specificities of “obtuse angle between cyst wall and mural nodule” and transmural invasion were comparable to that of findings suggestive of malignancy (Table 3). However, these MR findings had less sensitivity than findings suggestive of malignancy, although not significantly different. Findings suggestive of malignancy showed a maximum sensitivity of 83.3%, indicating that all SCC-MCT patients with mural nodules were malignant. Based on findings suggestive of malignancy, we were able to accurately diagnose three lesions without transmural invasion (Figs. 5, 6, 7).

## Discussion

While SCC-MCT pathologically presents as a nodule inside the cystic tumor or a plaque on the cyst wall [2, 7], no study has focused on the MR characteristics of mural nodules within SCC-MCTs. Herein, we explored characteristic MR findings of mural nodules in SCC-MCTs, comparing their diagnostic performance with that of the PRFs. Particularly, we focused on the solid components observed within MCTs and found that findings such as presence of diffusion restriction, absence of fat content, absence of Palm tree appearance, and absence of calcification were significantly more frequent in the SCC-MCT group. Furthermore, the four findings suggestive of malignancy were characterized by an excellent inter-reader agreement among radiologists, and they demonstrated higher diagnostic ability compared to the PRFs.

Projections called Rokitansky protuberances are often found in benign MCTs [8, 10]. Previously reported histological analysis of Rokitansky protuberances revealed the presence of sebaceous glands, adipose lobules, keratin, pilosebaceous adnexa, glial tissue, thyroid tissue, fibrous stroma, or vessels [15, 16]. Bone and teeth also tend to be observed in this protuberance and most of the hair arises from this nodule [3, 8, 13]. As a result, mural nodules within b-MCT often have calcifications and a Palm tree appearance. Rha et al. reported that a small fat component in the cystic wall of MCT can be detected using fat-saturated MR imaging or gradient-echo technique with both in-phase and opposed-phase imaging, which is useful to identify small amounts of fatty tissue on MR images [11]. Thus, the absence of these characteristic findings commonly observed in Rokitansky nodules is a distinctive feature of the mural nodules in SCC-MCT. However, as reported by Fujii et al., diffusion restriction in the mural nodules inside MCT, which often present as abnormal signal intensity on DWI in the diverse tissues of Rokitansky protuberances, does not alone indicate malignancy [17]. Moreover, squamous cell carcinomas typically exhibit diffusion restriction due to their high cellular density [18, 19]. Therefore, our study showed that diffusion restriction in the mural nodules alone does not rule out SCC-MCT; however, the absence of characteristic MR findings in the Rokitansky nodule (i.e., fat, calcification, and Palm tree appearance) combined with the occurrence of diffusion restriction in the mural nodule can lead to a diagnosis of SCC-MCT.

Several other useful findings to differentiate benign MCTs from malignant MCTs have been previously reported. The peak incidence of the MCT is reported to be between 20 and 40 years, while the mean age of malignant transformation is 45–60 years [3, 12]. The size of a malignant ovarian MCT is usually larger than that of a benign MCT [1–3, 5, 12, 20]. On MR images, SCC-MCT is characterized by transmural extension and infiltration into surrounding organs from solid components [7, 20, 21]. Furthermore, the analysis of CT and MR findings in 11 patients with this malignancy revealed that nine (82%) tumors had soft tissue components and eight (89%) had an obtuse angle between the soft tissue components and the inner wall of the cyst [2, 6]. Per findings of previous reports and the present study, the SCC-MCT group had significantly higher age, larger lesions, and greater mural nodule sizes compared to the b-MCT group. Additionally, mural nodules, obtuse angle between the nodule and the cyst wall, and transmural extension were observed significantly more frequently in the SCC-MCT group. These PRFs are considered important for differentiation between the two groups. However, if the mass is accompanied by transmural invasion, it is deemed to be already in the advanced stage of malignant transformation. Thus, imaging findings to detect SCC-MCT in its early stage are required. Additionally, there is a wide overlap in values of MR findings, such as tumor size, presence of mural nodules, and size of mural nodules, between benign MCT and SCC-MCT, making it difficult to establish clinically useful cut-off values [15, 16]. In the SCC-MCT group in our study, three cases did not exhibit transmural invasion, but conformed to the findings suggestive of malignancy (Figs. 5, 6, 7; Table 1). Additionally, two cases did not exhibit the PRFs for SCC-MCT, i.e., obtuse angle between the nodule and cyst wall. These three cases suggest the possibility that early-stage SCC-MCT can be diagnosed using findings suggestive of malignancy. In the Ovarian-Adnexal Reporting & Data System (O-RADS), a large volume of contrast-enhancing solid components in a lesion with lipid content presents an intermediate risk of malignancy [22]. However, it should be noted that even small mural nodules without transmural invasion may be indicative of malignancy. Therefore, diagnosis of SCC-MCT using findings suggestive of malignancy is crucial, regardless of patients’ age, tumor size, size of mural nodules, obtuse angle between the nodule and the cyst wall, or transmural invasion.

There were several limitations to our study. First, various MR imaging units and different protocols were included because this was a retrospective, long-term, and multicenter study. Second, while most institutions retain paraffin-embedded tissue blocks (PFFEs) for long durations, our retrospective study design presented specific challenges in correlating the mural nodules seen on MR images with their respective locations on pathology slides. This difficulty arises in retrospective analyses, where it is often not feasible to precisely match MR images with the exact sections in stored PFFEs. Consequently, we were unable to explore the correlations between the pathological findings and the radiographic findings suggestive of malignancy in the mural nodules. Third, there was a large difference in the number of cases between the benign and malignant groups, and it is necessary to take into account that there was a class imbalance bias in this study. However, it was unavoidable because SCC-MCTs are rare conditions. Finally, we did not investigate the malignant transformation of MCT that exhibit histological types other than SCC. This is because the number of cases with other histological types was extremely limited in the participating institutions. Therefore, it is unclear whether findings suggestive of malignancy can be applied to the malignant transformation to pathologies other than SCC.

In conclusion, characteristic MR findings of SCC-MCTs were as follows: mural nodules exhibited diffusion restriction, but, did not demonstrate fat content, Palm tree appearance, or calcification. These four findings suggestive of malignancy demonstrated higher diagnostic ability compared to the PRFs of age, tumor size, size of mural nodule, and the angle between mural nodule and cyst wall. Therefore, to provide accurate preoperative diagnosis of SCC-MCTs, cases with smaller mural nodules should be examined for the aforementioned four findings suggestive of malignancy.

## Data Availability

Not applicable.
